# Social and individual vulnerability factors associated with syphilis among populations living on islands in the Brazilian Amazon

**DOI:** 10.1186/s12879-023-08955-w

**Published:** 2024-01-02

**Authors:** Ellen Christiane Correa Pinho, José Jorge da Silva Galvão, Aline Maria Pereira Cruz Ramos, Cintia Yolette Urbano Pauxis Aben-Athar, Richardson Augusto Rosendo da Silva, Carlos Leonardo Figueiredo Cunha, Eliã Pinheiro Botelho, Glenda Roberta Oliveira Naiff Ferreira

**Affiliations:** 1 Programa de Pós Graduação Em Enfermagem, Federal, University of Para, Rua Augusto Correa, 01 – Setor Saúde, Guamá, Belém, Pará 66075-110 Brazil; 2 Universidade Federal Do Rio Grande Do Norte, Natal, Brazil

**Keywords:** Primary health care, Sexually transmitted infections, Syphilis, Prevalence

## Abstract

**Background:**

The repercussions of the syphilis epidemic differ according to populations. Identifying and acknowledging the differences and specificities of populations is fundamental in the design and implementation of policies aimed at assisting the groups most vulnerable to syphilis.

**Objective:**

To estimate the prevalence of antibodies against *Treponema pallidum* and associated vulnerability factors among riverside populations of a capital city in the Brazilian Amazon.

**Methods:**

Cross-sectional study was conducted among residents of the periurban islands in Belém, northern Brazil, from August 2020 to January 2021. The inclusion criterion was being a resident of the riverside communities of the Combú Environmental Protection Area, aged 18 years or over. The participants responded to questionnaire and were tested for syphilis using rapid test. Data were analyzed using multiple logistic regression by Minitab version 20® software.

**Results:**

Overall, a total of 325 riverine were included. Age varied from 18 to 91 years (average 40 years). Prevalence of markers for syphilis was 5.9% (95% CI: 3.3%-8.4%). The multiple regression showed that as age increases, the chances of having syphilis also increase (*p* = 0.001; aOR: 1.04) and riverside dwellers with more than one sexual partner in the last 6 months had more than four chances of having syphilis compared to people who had only one sexual partner (*p* = 0.007; aOR: 4.20).

**Conclusion:**

Syphilis circulates among traditional populations in the Amazon and is associated with factors of social and individual vulnerability.

## Background

Globally, syphilis cases was 30.91 million in 1990 and 49.71 million in 2019, with an increase of 60.83% in this period. Some regions and populations are disproportionately affected by the infection, mainly males and regions with low sociodemographic indices, such as in countries on the African continent, Latin America and the Caribbean [1]. In Brazil, there was an increase in the detection rate of acquired syphilis until 2018, with stability in 2019 and a decline in 2020 associated with the covid-19 pandemic. In 2022, 213,129 cases of acquired syphilis were reported (99,2 cases/100,000 inhabitants). The infection is also more prevalent in men and people with low education [2, 3].

Studies have shown that the syphilis epidemic and its repercussions differ according to populations [4–10]. Identifying and recognizing the differences and specificities of this process becomes essential in the design and implementation of policies aimed at assisting groups most vulnerable to syphilis [11–13]. In rural communities, the prevalence of syphilis in adults ranged from 0.2% among women in rural Nepal [5] to 16% among men living with HIV in rural Uganda [8]. Among populations living in seven fishing communities on Lake Victoria, in northwest Tanzania, the prevalence was 15.6% [9].

These are vulnerable populations in the social context and access to health services. There is a great lack of knowledge about Sexually Transmitted Infections (STIs), due to the low level of education and the absence of educational actions in health by the primary health care teams, which makes them very vulnerable to STIs [14, 15]. The riverside people, including people who live on the banks of rivers or on islands, have a way of life in which the geographic and environmental context of the place of residence alone is a structural barrier for them to have access to health services. To reduce this barrier, in the Brazilian Amazon, there are models of specific teams that work in Primary Health Care, riverside and river teams [15].

The prevention, diagnosis and treatment of syphilis actions are provided directly by these teams, without the need for referral to reference centers [16]. Due to the difficulty in accessing these communities, there are few studies in Brazil with riverine people [10, 17], of which only one was carried out in the Amazon, in the Marajó Archipelago, but also included populations urban areas of the cities that make up the Archipelago [17, 18]. This lack of studies makes it difficult to assess the population's access to actions aimed at syphilis that are offered by Primary Health Care. This is also due in part to the large number of compulsory syphilis notification forms that are not completely filled out by professionals [19]. In the Brazilian census, riverside dwellers are classified as rural populations [20]. Thus, making it difficult to design and evaluate public policies for these vulnerable populations. Given this scenario, this study aims to estimate the prevalence of antibodies against *Treponema pallidum* and associated vulnerability factors among riverside populations of a capital city in the Brazilian Amazon.

## Methods

### Study design

A cross-sectional study was conducted among residents of the periurban islands in Belém, northern Brazil, from August 2020 to January 2021.

### Setting

**Fig. 1:**
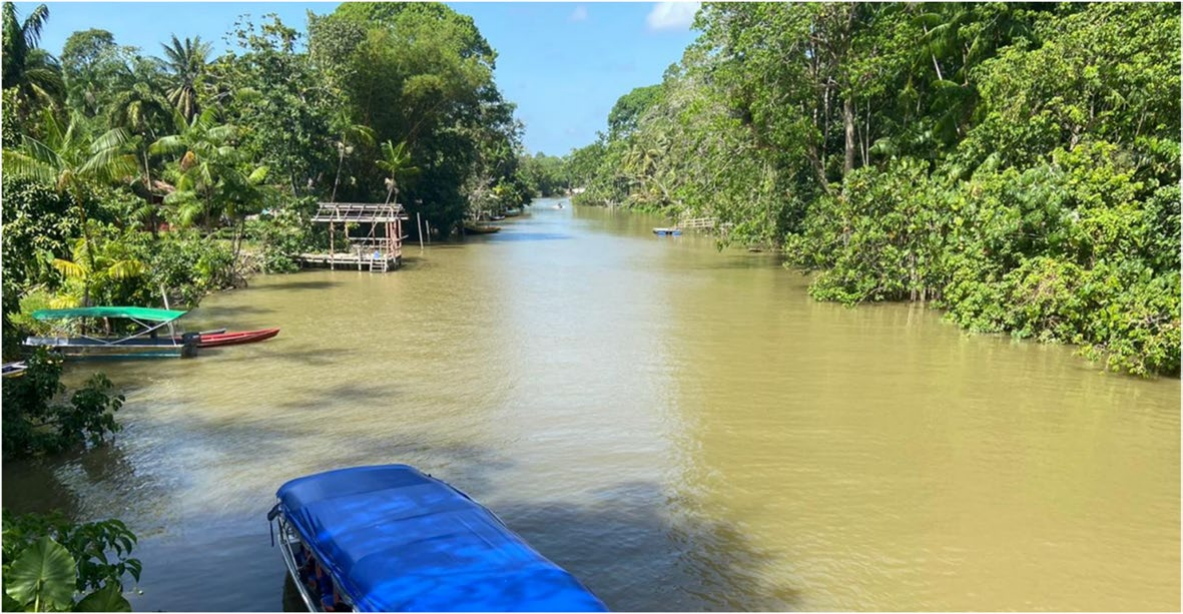
Combú Environmental Protection Area. Source: macroproject database

Belém is the capital of the state of Pará, in the brazilian Amazonian. A total of 11,294 people inhabit 39 islands in this city, one percent (1%) of the total population of the Belém. Combú Environmental Protection Area are periurban islands with about 2,200 inhabitants and area 14,770.000 square meters (Fig. 1). The riverine populations had precarious social and health indicators. There are public facilities provided by the government as one elementary school, one speedboat for school transport and healthcare, one basic health unit with one family health strategy team [15, 20–23].

**Fig. 2:**
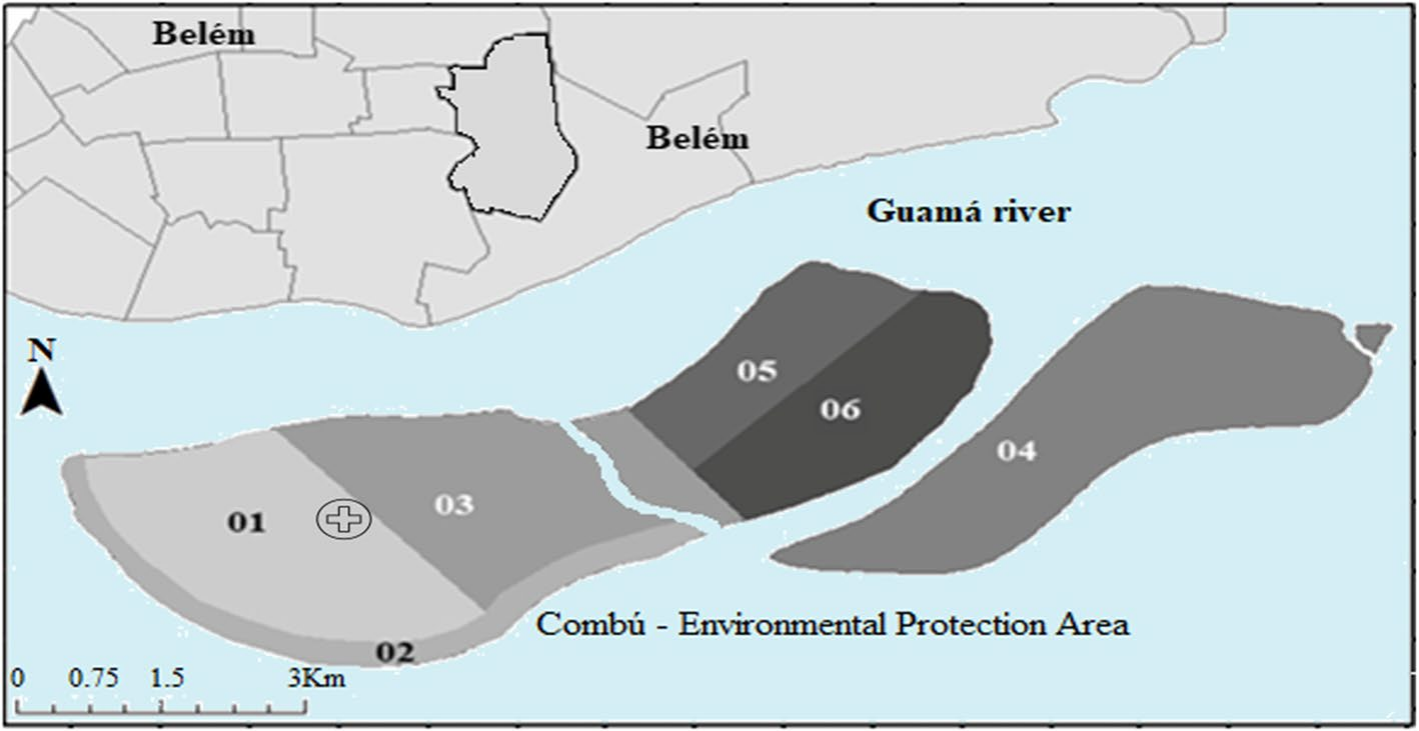
Geographic location of the island investigates in Belém, state of Pará, Brazilian Amazon. Legend: Micro areas: 1,2,3,4,5,6. symbol: location Combú's basic health unit. Source: Prepared by the authors, in the laboratory of the macroproject research group

The basic family health unit is responsible for six micro areas and is located in micro area 1, requiring the team to travel by boat to carry out care in the houses (Fig. 2).

### Participants

The inclusion criterion was being a resident of the riverside communities of the Combú Environmental Protection Area, aged 18 years or over. Those who were under the influence of psychotropic drugs or alcohol were excluded.

### Variables

The main hypothesis of the study was to analyze whether there is an association between vulnerability factors and a reactive result in the treponemal test (antibodies against *T. pallidum*). The dependent variable to test this hypothesis was the rapid test result for syphilis (treponemal test). This variable was treated as reactive and non-reactive, whereas the response event selected was reactive result (presence of antibodies against *T. pallidum* in the rapid test).

The independent variables were a) social: sex, age group, marital status, level of education, family income, skin color, number of people living in the house, beneficiaries of the social programs. B) individual: currently have a sexual partner, history of sexually transmitted infections in the last six months, sex with more than one partner in the last 6 months, condom use during recent sexual intercourse (last time), condom broken during sexual intercourse (at any time during life), frequency of condom use (last 3 months). C) programmatic: performed rapid test for Sexually Transmitted Infections—ever in life, Performed rapid test for Sexually Transmitted Infections (last 12 months), free access to condoms in the last 12 months, free access to female condom in the last 12 months, Know Post-Exposure Prophylaxis, Do you know female condom?

### Study size

The required sample size was calculated using a confidence interval of 97%; an expected prevalence of syphilis of 8.5% based on a previous study [17], a total of 320 participants. This total sample size was proportionally allocated for six micro-areas of the family health strategy team.

The simple random sampling technique wasn't employed to select the study participants due to the geographic conditions of the Combú island, with flooded areas accessible only by small boats. Thus, sampling was for convenience, at different times and days of the week to recruit people who work. Participants were recruited in the micro-area where they lived with the help of the community health agent responsible for the territory. It took 18 boat expeditions to reach the sample size.

### Data sources

The participants were asked to sign an informed consent if they were interested in the study and responded to questionnaire collected by trained researchers in individual and private face-to-face interviews. The instrument used was adapted from structured questionnaire “knowledge, attitudes and practices in the Brazilian population” [24]. This instrument was applied in a pre-test, with seven participants (not included in the study).

To assess prevalence of syphilis, after the interview, the participants were tested for syphilis using rapid test. They were invited to collect blood from the finger pulp of the hand for qualitative determination of total antibodies (IgG, IgM and IgA) against-*Treponema pallidum* (Kit Syphilis Bio—Bioclin, Minas Gerais, Brazil) [25]. In post-counseling, a trained nurse delivered the results to the participants.

Reactive results were complemented by Rapid Plasm Reagin test (RPR)—RPR Brás -Laborclin [26] performed from blood collected by venipuncture. For RPR, blood samples were processed by Virus Laboratory of the Institute for Biological Sciences, Federal University of Pará. The results were forwarded to the nurse, at the basic health unit on the island, for patient care and treatment when applicable.

### Statistical methods

Data were entered in the EPI Info version 7.2.2.16 (developed by the Centers for Diseases Control and Prevention in Atlanta, GA, USA) and exported to Microsoft Excel ®. For categorical variable if more than 20% of values are missed in one variable, we discard the variables. Descriptive statistics were made for categorical variables using absolute and relative frequencies. The result was presented using texts and tables.

The estimate of the prevalence of anti-*T. pallidum* and its confidence interval were calculated by estimating the proportion in the Bioestat 5.3® program. The main hypothesis of the study was tested using multiple logistic regression using Minitab software version 20®. Simple binomial regression was used to assess the association between each independent variable and the dependent variable. Variables with *p*-value < 0.20 (age group, Marital status, Level of education, Do you know female condom?, currently have a sexual partner, Sex with more than one partner in the past 6 months) were entered into a multiple logistic regression and the backward elimination method was applied. Age was analyzed in the regression as a continuous variable, being presented in the table by mean and by age group.

In which all variables are inserted into the regression and only the significant ones remain. For the interpretation of the results, adjusted Odd Ratios (aOR) with their respective p values and confidence interval (CI) were considered. The significance level adopted was 0.05.

## Results

Overall, a total of 325 riverine living in the Combú Environmental Protection Area were included. Age varied from 18 to 91 years (average 40 years). The majority of participant were in a relationship (Married/stable union/dating) at the time of data collection (70.1%; 228/325); had up to elementary education (56.6%; 184/325); with a monthly family income of less than one minimum wage (70.7%; 222/325) and are beneficiaries of social programs (66.4%; 216/325) (Table 1).

**Table 1:** Social factors associated with results for Syphilis among riverine, in the Brazilian Amazon. 2020–2021

**Social**	**Rapid test – *T. pallidum***		**Total** n (%)	**Binary Regression**		**Multiple regression**	
	**Non-reactive** n (%)	**Reactive** n (%)		**OR (95% CI)**	***p***	**aOR (95% CI)**	***p***
Sex							
Male	178(93.7)	12(6.3)	190(58.5)	Ref			
Female	128(94.8)	7(5.2)	135(41.5)	0.81 (0.31; 2.11)	0.85		
Age							
Mean (age)	39.3	52.1	40.0	1.04 (1.01; 1.07)	0.00	1.04 (1.01; 1.07)	0.00
Age group—18–41	187(96.9)	6(3.1)	193(60.5)				
Age group—42–65	71(93.4)	5(6.6)	76(23.8)				
Age group -Equal to or greater than 66	43(86.0)	7(14.0)	50(15.7)				
NI^a^	5	1	6				
Skin color							
Black	69(90.8)	7(9.2)	76(23.8)	2.13 (0.79; 5.72)	0.20		
White/brown/yellow	232(95.5)	11(4.5)	243(76.2)	Ref			
NI^a^	5	1	6				
Marital status							
Married/Stable union/Dating	218(95.6)	10(4.4)	228(70.2)	Ref			
Single/Divorced/Widowed	88(90.7)	9(9.3)	97(29.8)	2.22 (0.87;5.67)	0.09		0.19
Level of education							
High school/University	138(97.9)	3(2.1)	141(43.4)	Ref			
Never attended school/Elementary	168(91.3)	16(8.7)	184(56.6)	4.38 (1.25; 15.3)	0.02		0.29
Family income (minimum wage)^b^							
Up to one	211(95.0)	11(5.0)	222(70.7)	0.54 (0.21; 1.40)	0.31		
Equal to or greater than one	84(91.3)	8(8.7)	92(29.3)	Ref			
NI^a^	11	0	11				
Number of people living in the house							
Up to two people	80(96.4)	3(3.6)	83(25.5)	1.88 (0.53; 6.65)	0.46		
Equal to or greater than three	226(93.4)	16(6.6)	242(74.5)	Ref			
Participate in social programs (beneficiaries of the social programs)							
No	100(91.7)	9(8.3)	109(33.5)	0.53 (0.21; 1.36)	0.28		
Yes	206(95.4)	10(4.6)	216(66.5)	Ref			

*OR* odds ratio, *CI* confidence intervals, *Ref.* reference, *aOR* adjusted odds ratio

^a^NI: not informed/do not want/do not know—not considered for statistical calculation

^b^Brazilian monthly minimum wage 2020—BRL 1,045.00 per month

In the rapid test, the prevalence of antibodies against *T. pallidum* was 5.9% (19/325; 95% CI 3.3%-8.4%) in this study. After all samples were tested in the rapid test for *T. pallidum*, the reactive samples were tested in the RPR. One (0.3%; 1/325) participant had title equal to 1:8, confirmed diagnosis of syphilis using RPR. Titers less than or equal to 1: 4 were found in 3.4% (11/325) participants.

The social aspects associated with antibodies against *T. pallidum* among riverine are shown in Table 1. The analysis of the association of age in years (continuous variable) demonstrated that the chances of having a reactive result for syphilis increase with age (OR: 1.04; *p* = 0.002). Participants with primary education/never attended school are four times more likely to have reactive syphilis (OR: 4.38;* p* = 0.02). In addition to these two variables, also, the variable marital status with *p* < *0.20* was selected for the multiple regression.

Social factors are shown in Table 2. Among access to health services factors, only one variable was selected for multiple regression (*p* < 0.20): knowledge about the female condom.

**Table 2:** Access to health services factors associated with results for Syphilis among riverine, in the Brazilian Amazon. 2020–2021

**Access to health services**	**Rapid test – *T. pallidum***		**Total** n (%)	**Binary Regression**		**Multiple regression**	
	**Non-reactive** n (%)	**Reactive** n (%)		**OR (95% CI)**	***p***	**aOR (95% CI)**	***p***
Performed rapid test for STI (ever in life)							
No	140(95.9)	6(4.1)	146(46.9)	0.60 (0.21; 1.66)	0.45		
Yes	154(93.3)	11(6.7)	165(53.1)	Ref			
NI^a^	12	2	14				
Performed rapid test for STI (last 12 months)							
No	215(95.1)	11(4.9)	226(72.9)	0.66 (0.23; 1.85)	0.61		
Yes	78(92.9)	6(7.1)	84(27.1)	Ref			
NI^a^	13	2	15				
Access to condoms (12 months)							
No	133 (93.7)	9 (6.3)	142 (44.5)	1.37 (0.47; 3.97)	0.74		
Yes, I bought it in a commercial establishment	45 (91.8)	4 (8.2)	49 (15.4)	1.80 (0.48; 6.70)	0.59		
Yes, free in actions and in the health service	122(95.3)	6(4.7)	128 (40.1)	Ref			
NI^a^	5	0	6				
Access to female condom (last 6 months)							
No	271 (93.8)	18 (6.2)	289 (88.9)	2.32 (0.30; 17.9)	0.64		
Yes, free in the health service	35(97.2)	1(2.8)	36 (11.1)	Ref			
Know Post-Exposure Prophylaxis							
No	280(94.3)	17(5.7)	297(91.4)	0.78 (0.17; 3.60)	0.90		
Yes	26(92.9)	2(7.1)	28(8.6)	Ref			
Do you know female condom?							
No	77(89.5)	9(10.5)	86(26.8)	3.44 (1.03; 11.6)	0.06		0.24
Yes, professionals and/or the media	225	10	235	Ref			
NI^a^	4	0	4				

*STI* Sexually Transmitted Infections, *OR* odds ratio, *CI* confidence intervals, *Ref.* reference, *aOR* adjusted odds ratio

^a^ NI: not informed/do not want/do not know—not considered for statistical calculation

Among the individual factors (Table 3), the following variables were selected for multiple regression (*p* < 0.20): Have currently have a sexual partner (*p* = 0.05) and sex with more than one partner in the past 6 months (OR: 2.94; *p* = 0.03).

**Table 3:** Individual factors associated with results for Syphilis among riverine, in the Brazilian Amazon. 2020–2021

**Individual**	**Rapid test – *T. pallidum***		**Total** n (%)	**Binary Regression**		**Multiple regression**	
	**Non-reactive Reactive** n (%)			**OR (95% CI)**	***p***	**aOR (95% CI)**	***p***
Currently have a sexual partner							
No	62(88.6)	8(11.4)	70(21.5)	2.86 (1.10; 7.41)	0.05		0.59
Yes	244(95.7)	11(4.3)	255(78.5)	Ref			
History of STI (last 6 months)							
No	29(96.7)	1(3.3)	30(9.2)	Ref			
Yes	277(93.9)	18(6.1)	295(90.8)	1.88 (0.24; 14.6)	0.83		
Sex with more than one partner in the past 6 months							
No	252(95.5)	12(4.5)	264(82.2)	Ref			
Yes	50(87.7)	7(12.3)	57(17.8)	2.94 (1.10; 7.83)	0.03	4.20 (1.46; 12.05)	0.007
NI^a^	4		4				
Frequency of condom use (last 3 months)							
Never	164(94.3)	10(5.7)	174(57.4)	0.79 (0.20; 3.01)	0.98		
Sometimes	82(94.3)	5(5.7)	87(28.7)	0.76 (0.17; 3.36)	0.97		
Everytime	39(92.9)	3(7.1)	42(13.9)	Ref			
NI^a^	21	1	22				
Condom use during recent sexual intercourse (last time)							
No	207(94.5)	12(5.5)	219(68.7)	0.77 (0.29; 2.01)	0.78		
Yes	93(93.0)	7(7.0)	100(31.3)	Ref			
NI^a^	6	0	6				
Broken condom (at any time during life)							
No	218(94.0)	14(6.0)	232(73.7)	Ref			
Yes	78(94.0)	5(6.0)	83(26.3)	0.99 (0.34; 2.86)	0.79		
NI^a^	10	0	10				

*STI* Sexually Transmitted Infections, *OR* odds ratio, *CI* confidence intervals, *Ref.* reference, *aOR* adjusted odds ratio

^a^ NI: not informed/do not want/do not know—not considered for statistical calculation

Variables with *p*-value < 0.20 (age group, Marital status, Level of education, Do you know female condom?, currently have a sexual partner, sex with more than one partner in the past 6 months) were entered into a multiple logistic regression and the backward elimination method was applied.

The multiple regression showed that as age increases the chances of having syphilis also increase (*p* = 0.001; OR: 1.04) and riverside dwellers with more than one sexual partner in the last 6 months had more than four chances of having syphilis compared to people who had only one sexual partner (*p* = 0.007; OR: 4.20).

## Discussion

The estimated prevalence of antibodies *against T.pallidum* among the riverside population of a capital in the Brazilian Amazon, Belém, was 5.9%, with social and individual vulnerability factors associated with the presence of infection markers. Active syphilis had a low frequency among participants. In this community, which has a family health strategy team, no association was found between vulnerability factors related to access to health services and syphilis.

The North region, Brazilian Amazon, had the second lowest detection rate of acquired syphilis in 2022, with 86.3 cases per 100,000 inhabitants (16,518 cases), behind only the Northeast region with 55.4 cases per 100,000 (32,084 cases) [2]. Studies carried out in Brazil found a lower prevalence than the present study, such as among quilombola women (another traditional population) where the prevalence was 4.3% by rapid test [27] and among sugarcane cutters sugar, serological markers of lifelong syphilis were detected in 2.5% (by rapid treponemic test) and active syphilis in 1.2% [4]. However, a higher prevalence than the present study was found among riverside dwellers who do not inhabit islands, in the state of Paraíba in the Northeast region, where the prevalence was 11.6% by rapid treponemic test [10] and among the inhabitants of the largest river island in the country Marajó Island, in Pará, the prevalence was 8.5% by immunoenzymatic assay, ELISA type [17].

In the present study, the final logistic regression model demonstrated that the factors associated with antibodies against *T. pallidum* were having sex with more than one partner in the last six months and older age. Among riverside dwellers in Paraíba, the number of partners was a factor associated with syphilis (more than two sexual partners in the last 12 months, *p* = 0.005), along with two other factors, previous history of STIs (*p* < 0.001) and history of imprisonment (*p* = 0.010) [10].

This result is related to the type of antibodies detected in the rapid test used, as it detects antibodies from past infection. Therefore, older age allows for a longer period of exposure to bacteria when prevention methods against infection are not used. In Brazil, in basic primary care health units in the single health system, reverse testing is used to diagnose syphilis, which consists of performing a rapid treponemal test to detect antibodies against *T. pallidum* of the IgG, IgA and IgM classes. The reagent results are subjected to a second test to detect active infection [2, 16].

Among women in rural areas of China, multiple analysis of sociodemographic factors identified that older age is associated with a reactive result for syphilis, along with other factors such as low education (elementary or lower), being from ethnic minorities and of specific provinces. In the multiple analysis of obstetric and sexual history, an association was demonstrated with markers for *T. pallidum*, having a history of pregnancy and STI or gynecological disease, women who never used condoms and those with husbands who tested positive for syphilis [28]. The number of partners and age were not factors associated with syphilis among people in rural areas of Ghana. In this population, significant factors associated with syphilis infection included sub-district of residence, and history of coerced sexual intercourse [29].

In studies carried out in the general population, the number of sexual partners was among the factors identified in the multiple regression [30, 31]. The number of sexual partners in the last year (three or more) was also shown to be a factor for gestational syphilis among women in maternity wards in a city in the Northeast region of Brazil [30]. Among blood donors in Chengdu, China, having two or more partners was associated with syphilis along with factors other than age [31].

These studies demonstrate that, along with other factors, the number of partners is an important factor in increasing exposure to *T. pallidum* [10, 28–31]. The recognition of the profile or vulnerability index of a population makes it possible to target specific strategies for each population [27–36]. Brazil has a national health system with universal access to all levels of health care that has been able to improve the health indicators of the most vulnerable populations, despite the great social inequality that still persists. More recently, the COVID-19 pandemic has demonstrated this importance and the need for public policies aimed at reducing social inequalities [32, 37].

In Brazil, the rapid test for syphilis should be offered in all basic health units, being an important screening strategy for remote populations, such as in the Amazon. The nurse is legally able to request the test, perform it and issue the report, but there is still uncertainty when delivering a reactive result [12]. For the riverside population studied, having a family health strategy team facilitates access to diagnosis and treatment, as well as actions aimed at prevention. Techno-assistance models riverine and river family health teams were strategies that are present in Brazil's National Primary Care Policy that led to the inclusion of a population that is dispersed over large areas of the municipalities' territory, consequently, they can expand access to HIV screening. syphilis and start treatment to interrupt the chain of transmission [16, 38].

Health professionals who work in these teams receive free training aimed at reducing syphilis. In Brazil, between February 2019 and September 2020, free training and lifelong learning strategies were offered to health professionals aimed at reducing syphilis, with about 22,000 students participating [39]. A previous study has already shown that the increase in the number of nurses was significantly associated with chronic diseases [40]. In this way, it may be able to minimize the vulnerability of access to health services, as observed in the study.

Among the limitations of the study is the sample calculation that was carried out using the number of inhabitants of the island aged 18 or over as a population parameter, as well as the sampling method was non-probabilistic. Another limitation was the cross-sectional observational design, which cannot establish a cause and effect relationship between the factors and syphilis, since a cohort was not carried out. The study did not address other (behavioral) variables that could be associated with syphilis. Generalizations must be made considering the techniques used in the analysis, in the detection of infection markers and in the population studied.

## Conclusions

The results of the study demonstrate that the bacteria that causes syphilis circulates among the riverside population of the Brazilian Amazon. Social and individual factors associated with infection markers made it possible to understand the vulnerability profile of this population, which is important for directing specific care, such as health education on the transmission and prevention methods of syphilis for the young and adult population. In this context, it is important to detect the cultural aspects and lifestyle habits of the population.

## Data Availability

The datasets used during this current study are also available from the corresponding author on reasonable request.
